# Investing to improve conditions for retention and satisfaction at a paediatric eye centre in South Sulawesi, Indonesia

**Published:** 2018-07-31

**Authors:** Marliyanti Nur Rahmah Akib

**Affiliations:** 1 Paediatric ophthalmologist and Paediatric Eye Centre Coordinator: Hasanuddin University Hospital, Makassar, Indonesia.

Increasing the capacity and retention of human resources in specialised tertiary health centres is very important in order to achieve good quality eye care services for children. Our paediatric eye centre, part of the university hospital of Hasanuddin in Makassar (a province of South Sulawesi), provides services to the eastern islands of Indonesia.

Until recently, our centre was led by a paediatric ophthalmologist and a refractionist. At that time, the number of children we were able to help was very low, as refractions, orthoptic evaluations, ocular examinations and counselling took a long time, which meant that children and their parents had to wait for extended periods. Low vision assessment was similarly affected. The working conditions were stressful and staff members' overall satisfaction levels were low.

In 2016, supported by the Seeing is Believing programme and collaborating partners, a significant investment was made to increase the number of trained staff responsible for providing paediatric services. In addition to the paediatric ophthalmologist and refractionist, the centre now has a second ophthalmologist, an orthoptist, a counsellor, a low vision specialist and a rehabilitation worker. The centre was moved to a more child-friendly environment and the flow of patients through the clinic was re-organised to reduce waiting times and improve the quality of services.

**Figure:**
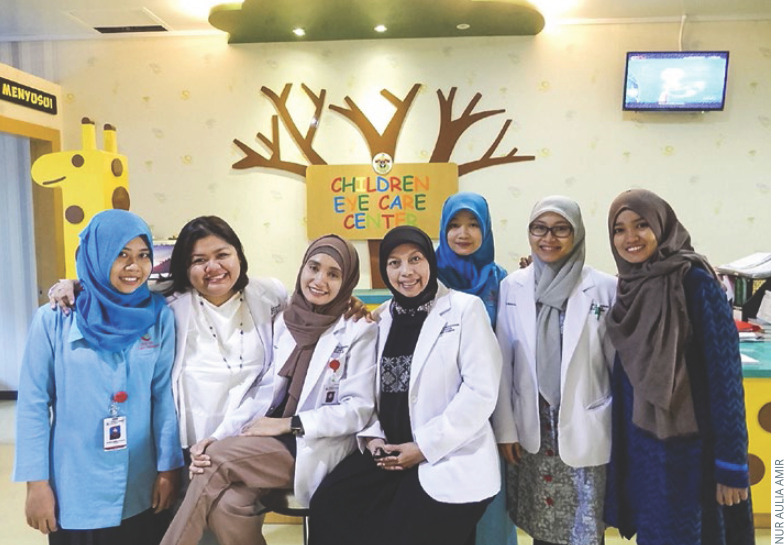
Improving conditions in an eye unit benefits staff and the community. INDONESIA

Since this additional investment, the waiting time for patients in the outpatient department has decreased by 75% and up to 20 patients are seen daily. Furthermore, surgical referrals have become more varied as the ophthalmologists are now able to handle complicated cases. Low vision services improved after staff members at the clinic received basic and advanced low vision training, which is supported by the new facilities and space available to them. Currently, 3–5 paediatric low vision cases are assessed each day, compared to 1–2 cases per month before.

Retaining our new staff members is very important to us. In addition to the services they provide in the eye clinic, we encourage our team to take on the role of teaching residents and/or being responsible for outreach activities, including school eye health screening. We also encourage them to discuss patients' cases in internal meetings. All of this gives them a sense of belonging in the team and in the wider community. In order to give our staff members the best financial package we can, we now employ them as civil servants.

*With thanks to Marliyanti Akib, Adelina Poli, Abrar Ismail, Habibah Muhiddin, Rishiraj Borah and Satyaprabha Kotha*

